# When and how do individuals transition from regular drug use to injection drug use in Uganda? Findings from a rapid assessment

**DOI:** 10.1186/s12954-019-0350-2

**Published:** 2019-12-23

**Authors:** Matayo Baluku, Twaibu Wamala

**Affiliations:** 1 Infectious Diseases Institute, Makerere University, College of Health Sciences, Kampala, Uganda; 2 Uganda Harm Reduction Network, Kampala, Uganda

**Keywords:** People who inject drugs, Hepatitis C Virus, HIV, Transition, Harm reduction, Uganda

## Abstract

**Background:**

In Uganda, injection drug use is a growing but less studied problem. Preventing the transition to injection drug use may help prevent blood-borne viral transmission, but little is known about when and how people transition to injection drug use. A greater understanding of this transition process may aid in the country’s efforts to prevent the continued growth of injection drug use, HIV, and hepatitis C Virus (HCV) infection among people who inject drugs (PWID).

**Methods:**

Using a rapid situation assessment framework, we conducted semi-structured interviews among 125 PWID (102 males and 23 females)—recruited through outreach and snow-ball sampling. Participants were interviewed about their experiences on when and how they transitioned into injection drug use and these issues were also discussed in 12 focus groups held with the participants.

**Results:**

All the study participants started their drug use career with non-injecting forms including chewing, smoking, and sniffing before transitioning to injecting. Transitioning was generally described as a peer-driven and socially learnt behavior. The participants’ social networks and accessibility to injectable drugs on the market and among close friends influenced the time lag between first regular drug use and first injecting—which took an average of 4.5 years. By the age of 24, at least 81.6% (95.7% for females and 78.4% for males) had transitioned into injecting. Over 84.8% shared injecting equipment during their first injection, 47.2% started injecting because a close friend was already injecting, 26.4% desired to achieve a greater “high” (26.4%) which could reflect drug-tolerance, and 12% out of curiosity.

**Conclusions:**

Over 81% non-injecting drug users in Kampala and Mbale districts transitioned into injecting by the age of 24; a process that reproduces a population of PWID but also puts them at increased risk of HIV and HCV infection. As Uganda makes efforts to introduce and/or strengthen harm reduction services, interventions targeting non-injecting drug users before they transition into injecting should be considered as a key component for HIV/HCV epidemic control efforts, and their evaluation considered in future researches.

## Introduction

Non-medical use of drugs is an epidemic in many parts of the worl d[1–4]. They are taken into the human body in a wide variety of ways, including smoking, snorting or sniffing powder or solution (intranasal use), inhalation of the heated vapors (“chasing”), orally, and as anal suppositories (“plugging”) or injected [5–7].

In 2016, it is estimated that 275 million people, which is roughly 5.6% of the global population aged 15–64 years, used drugs at least once. Of these, 10.6 million (range 8.3 million to 14.7 million) were people who inject drugs (PWID) [3]. While all methods of using drugs can be linked with social and health harms, injecting—whether intravenous, subcutaneous, or intramuscular, is the method of administration carrying the highest risk for multiple types of infections, overdoses, and their complications [5, 8–11]. Injecting drugs also carries high risk of human Immunodeficiency virus (HIV) and viral hepatitis transmission if sterile injecting equipment is not easily accessible and injecting equipment is shared among users. In 2016, more than half of the PWID worldwide were living with hepatitis C virus (HCV), one in eight were living with HIV, and 82.4% of the PWID living with HIV were co-infected with HCV [12–16]. The risk of acquiring HIV for PWID in 2017 was 22 times higher than that for people who did not inject drugs [17].

In Uganda, there is limited data on PWID. In 2017, the estimated number of PWID in Kampala was 3892 (3090–5126) [18]. The HIV prevalence among PWID is estimated at 45% compared to 15% in Kenya and 20% in Tanzania [19–23]. In their study in two urban centers of Kampala, the capital of Uganda and Mbale Municipality, Baluku et al. established that the median age at first injection was 19. While only 15.2% of PWID start injecting before the age of 17, 81.6% reported injecting by the age of 25. The drugs commonly injected included brown heroin (44.8%), white heroin (30.4%), and cocaine (16%) [16, 24, 25]. Many non-injecting drug users may be at risk of transitioning to injecting drug use, increasing their risk of acquiring or transmitting blood-borne infections. Moreover, transitions to injecting among infected non-injecting drug users may increase the number of infected PWID and further the spread of these pathogens among the injecting community and to the general populations through their sexual networks. It thus precipitates a cascade of negative health outcomes [26, 27]. Given these serious health consequences, this project aimed at assessing transitions to injecting among non-injecting drug users and the risk factors for transition in Uganda. Such information is essential to preventing transition to injection, which is the most effective way of preventing injection-driven HIV epidemics [28, 29].

Additionally, in Uganda, there is a call to and indeed some efforts are being put in place for introduction of harm reduction services for PWID as part of the efforts to control the HIV epidemic [16, 30, 31]. However, there are limited strategies available to support the people who use *but do not inject* drugs. Support for this sub-population, and in particular supporting the prevention of transitions to injecting drug use is, therefore, a potentially key priority for HIV prevention. In this paper, we provide insights from a rapid situation assessment to address key gaps in available knowledge on injection initiation processes, how regular drug users transition into injecting drug users in Uganda, and how these can be used to integrate harm reduction services targeting non-injecting drug users before they transition into injecting as a key component in HIV and HCV epidemic control efforts.

## Methods

The study was a rapid situation assessment (RSA) [32]. It was conducted from February to September 2017 as part of a broader study to establish the current situation of injecting drug use in Uganda and identify the country specific links between drug use and HIV and HCV risk, that are in part described elsewhere [16, 22, 33].

The study was conducted in two urban centers: Kampala City and Mbale Municipality. Kampala is the capital and business center of Uganda. It was selected on purpose because it is thought to have the largest number of people who inject drugs in Uganda [18]. Mbale Municipality, on the other hand, is one of the largest and busiest towns in eastern Uganda, but also a transit route from neighboring Kenya. It is a blossoming urban center [34].

We used an inductive approach [35, 36] during this rapid assessment, whereby initial data collected informed planning and decisions about subsequent data collection. The rationale for this approach was to allow the research team to make sense of the data collected, determine its utility and adequacy, identify gaps, and then collect additional data if necessary.

The inclusion criteria for the study included aged 18 years and above, having injected drugs for non-medical purposes at least once in the past three months, resident within the two study sites, and ability to voluntarily consent to take part in the study. Persons who were deemed to be “high” on drugs at the time of contact were re-scheduled to be interviewed at another time, and if that was not possible, they were excluded.

A total of 125 PWID (95 in Kampala and 30 in Mbale) recruited through snowball sampling [37] and direct recruitment at drug hotspots participated in the study. Data on drug-related behaviors and practices at initiation of drug use and transition to injecting were collected from them using semi-structured interviews. These issues were also discussed in 12 focus groups held with the participants. Seven of the focus group discussions (FGDs) were done in Kampala and five in Mbale. Each of the FGD consisted of 6–10 participants. The FGDs also explored how they started using drugs, the type of drugs they used, the circumstances that interested the participants to start injecting, and the time lag from regular drug use to injecting. We also inquired on their experience on the first injection including their age at first injection, drugs they used at first injection, and whether or not the shared injecting equipment at first injection. The broader study, including the detailed methods, are described in full elsewhere [16, 22, 33].

Quantitative data collected were analyzed using MS Excel and described in terms of frequencies and percentages on the different variables. We adopted the thematic analysis approach described by Braun and Clark [38] to analyze the qualitative data progressively as it was collected. It generally followed the steps outlined: organizing and ordering the raw data, coding the interviews, listing and sorting the data, categorizing and summarizing the data, interpreting the data, triangulating, validating, and drawing conclusions. The information was classified according to themes and domains and presented in form of taxonomies that reveal emerging patterns.

## Results

### Demographic characteristics of the sample

A total of 125 PWID were recruited into the study. Of these, 102 (81.6%) were males and 23 (18.4%) were females (18.4%). The mean age was 27.4 (females, 25.9 and males, 27.8). Only 30.4% of the participants were married or living together. At least 71 (56.8%) participants had attained a minimum of secondary education, and 17 (13.6%) earned their living through sex work. Table 1 has details of the demographic characteristics of the participants, stratified by study area.

**Table 1:** Demographic characteristics of the participants

Characteristic	Kampala	Mbale
	Number (%)	Number (%)
Biological sex of participant		
Male	77 (81.1)	25 (83.3)
Female	18 (18.9)	5 (16.7)
Age of participant		
18–24	32 (33.7)	12 (40.0)
25–29	35 (36.8)	16 (36.7)
30–34	15 (15.8)	3 (10.0)
35 and above	13 (13.7)	4 (13.3)
Level of education		
No education	3 (3.2)	0 (0.0)
Primary incomplete	19 (20.0)	7 (23.3)
Completed primary education	23 (24.2)	2 (6.7)
Secondary	34 (35.8)	16 (53.3)
Post-Secondary	16 (16.8)	5 (16.7)

### The processes of transitioning

The PWID who participated in the study indicated that they started drug use with non-injecting forms of drug consumption such as chewing, smoking, and sniffing of drugs. They also indicated that they usually started with “cheaper and simple” drugs such as *marijuana* and *kuba*, before upgrading to “expensive and hard” drugs such as cocaine and heroin. Most of the participants used other non-injecting forms of cocaine and heroin before they started injecting them. However, they were those that transitioned from the “cheaper and simple” drugs directly to injecting heroin and cocaine. They emphasized that each one’s journey into injecting varied with the circumstances that surrounded the individual. Once the decision to inject had been reached, they were often helped by friends on their first injection until they got used and started injecting themselves.

R1: You start by going with your friends where they inject from and you see how they are injecting. R2: In the ghettos they do both injecting and smoking, so you start by smoking and with time you also start injecting. Injecting is not easy so you cannot just start like that. R3: You start by chewing, then smoking and then injecting. R5: You do not inject yourself in the first days of learning, you ask your friends to help you. When you get used then you start injecting yourself (FGD, FSW PWID, Kabalagala, Kampala).

We didn’t start with injections, I started with smoking weed or marijuana and as time went on, I graduated to more effective and strong drugs and eventually started doing injections. I started with marijuana then moved to heroin powder and then cocaine and coke (FGD, PWID, Bakuli, Kampala).

R10: I started by sniffing brown sugar [an adulterated or impure form of heroin] then moved to injecting. R4: I started by smoking marijuana then moved to inhaling brown sugar, smoking, then to injecting. R3: I have cousins from South Africa who started injecting brown sugar. While for me I was scared. So, I used to sniff brown sugar and kent [concoction of local leaves and Catha edulis], when they returned I had gotten courage to also face the injection! R5: I started with kent because you can sniff it, later on my boyfriend told me it would damage my brain so he started injecting me with snooze instead! (FGD, MSM PWID, Mbale).

### Behaviors for PWID at initiation of drug use and transitioning to injecting

Fifty-two percent of the respondents had initiated drug use before the age of 17. More males (57%) started using drugs before the age of 17 than the females (33.3%). Table 2 has details on the participants’ age at first drug use and injecting.

**Table 2:** Age at first drug use and injecting

Characteristic	Female	Male
	Number (%)	Number (%)
Age at first drug use		
10–17	7 (33.3)	57 (55.9)
18–24	13 (61.9)	39 (38.2)
25 and above	1 (4.8)	6 (5.9)
Age at first injection		
10–17	5 (21.7)	14 (13.7)
18–24	17 (73.9)	66 (64.7)
25 and above	1 (4.3)	22 (21.6)

The drugs first used by the PWID in the study area included alcohol (89.6%), *khat* (77.6%), and cannabis (77.6%). None of the participants initiated drug use through injecting.

On another hand, by the age of 17, at least 15.2% of the respondents had transitioned into injecting. More females (21.7%) than males (13.7%) transitioned by the age of 17; and by the age of 24, at least 81.6% of the respondents (95.7% for females and 78.4% for males) had transitioned. Table 3 has details of the drug-use behaviors for the participants at transition.

**Table 3:** Drug-use behaviors for PWID at initiation and transition

Type of behavior	Value
Age of first drug use (median)	15
Age of first injection (median)	19
Interval between first drug use and first injection (mean)	4.5
The types of first drug used (*n*(%))^a^	
Cannabis	86 (68.8)
Alcohol	112 (89.6)
*Khat*	97 (77.6)
*Kuba*	42 (33.6)
Fuel (sniffing)	8 (6.4)
Heroin	5 (4.0)
Cocaine	26 (20.8)
The types of first drug injected, *n*(%)	
Brown heroin	76 (60.8)
White heroin	29 (23.2)
Cocaine	17 (13.6)
Others	3 (2.4)
Place of first injection, *n*(%)	
Ghetho/dungeon/hideout	78 (62.4)
My home	3 (2.4)
Supplier’s home/premise	26 (20.8)
Friend’s home	11 (8.8)
Others	7 (5.6)
Sharing practice	
Shared injecting equipment during first injection	106 (84.8)
Reasons for transitioning	
My friends were injecting	59 (47.2)
To achieve a greater/more effective high	33 (26.4)
Curiosity	15 (12)
Other	18 (14.4)

^a^Multiple options were available due to polypharmacy

On average, it took 4.5 years for participants to transition from the first drug use to their first injection use. While there were individuals who knew a few friends that had abandoned injecting after the first injection, majority of the respondents continued with the injecting behavior—with the second and third injection happening almost immediately after the first.

Qualitative data revealed that the period between regular non-injecting use of drugs and first injecting was very dependent on the participants’ social networks, and accessibility to injectable drugs, both on the market and among friends. If one had close “injecting friends” with regular supply of drugs, they had higher chances of transitioning early.

If your peers are mainly people who inject, you can even start injecting within six or less months FGD, Mbale.

This transition period varies significantly from individual to individual. Most of the PWID we interact with transitioned within one to two years...it doesn’t mean that one can’t take over five years…as drugs continue to be more accessible on the market, this period might shorten PWID Peer, Kampala

Majority of my babulaadi [blood brothers] were already injecting…so I had access to those to help [me] to start injecting but also I could share their drugs and syringes FGD, Bwaise.

The most common drug used for first injection was brown heroin reported by 60.8% and white heroin (23.2%). Some respondents reported that other times they are not aware of the type of drug they are injecting; or it is a mixture without a known name.

I really don’t know the names…. it’s my “charlie” [friend] who mixes for me… and I just inject (FGD, FSW PWID, Mbale)

Yes! We mix kuba, taaba [local tobacco] and aviation fuel, then boil the mixture, cool it, filter it then inject. After injecting it, one feels they are in heaven (FGD, FSW PWID, Mbale)

Sharing of injection equipment was reported by participants. At least 106 (84.8%) shared needles and syringes directly. Other forms of indirect sharing, on first injection, that were elicited during FGDs included drawing up the dissolved drug from a solution shared by others and sharing of injecting paraphernalia like cotton and filters.

### The reasons for transitioning into injecting drug use

Nearly one-half of the participants started injecting because they had an “injecting-friend.” There was a popular belief that PWID were “better in status” than their non-injecting peers. They indicated that starting to inject would make the non-injecting friends relate better with their injecting peers. The participants also pointed out that the non-injecting friends admired their injecting colleagues because injecting seemed to make them feel “better and high” compared to other forms of drugs like smoking. More than one-quarter of the participants switched to injecting in order to get more joy or high.

Qualitative data also pointed out that peer influence was a strong factor in the initiation of drug use generally and transitioning into injecting drug use, and many reported that they learnt from and were influenced by friends.

R2: You start by going with your friends where they inject from and you see how they are injecting. R1: Injecting is not easy so you cannot just start like that. R4: Our partners also lead us to start. R5: You do not inject yourself in the first days of learning, you ask your friends to help you. When you get used then you start injecting yourself (Female Sex Workers’ FGD PWID, Kabalagala, Kampala)

R5: I started with kent because you can sniff it, later on my boyfriend told me it would damage my brain so he started injecting me with ‘snooze’ [snus, a tobacco-based product] instead! (Men who have sex with men’s FGD, PWID, Mbale).

To prove a sense of belonging and being curious also emerged as reasons for transitioning also emerged from the qualitative data.

R3: Some just want to find out how it feels like when you inject. R6: Others want to fit in the society. R7: Others just want to share injection with you to show that they love you so much, …we are one by blood and heart (FGD, FSW PWID, Kabalagala, Kampala)

## Discussion

This study is the first to report behaviors surrounding transitioning from regular drug use to injecting drug use among PWID in Kampala Capital City and Mbale Municipality, Uganda. Overall, it shows that drug use in the study area is a phenomenon that starts during adolescence with more than one-half of the participants having started to use the drugs before the age of 17. Similarly, while the study shows that injecting drug use also starts during adolescence, only 15.2% of the participants had transitioned into injecting by the age of 17. By the age of 24, at least 81.6% of the participants had transitioned into injecting. More females (95.7%) than males (78.4%) transitioned before the age of 24. Qualitative findings indicated that women were likely to be influenced easily into injecting by their male peers and clients especially if they engaged in sex work. This suggests, in part, that adolescent girls and young women who inject drugs in the study area are likely to transition earlier than their male counterparts. Studies elsewhere have also established that transitioning, like other injecting drug practices, have significant gender differences [39–41]. Therefore, as Uganda plans to introduce harm reduction policies and programs, they should be sensitive the local gender dynamics.

The mean age at first injection of 19 established by this study is comparable to that of many other countries in which the age of first injection varies from 19 to 22 years [42–45]. It, however, contrasts with other studies which have found older ages of first injection drug use for up to 29.87 ± 6.54, for example in Iran [46, 47]. As such, harm reduction services in Uganda including prevention of transitioning should focus on young people.

Also, the time lag from the first drug use and first injection was 4.5 years. This is relatively short compared to other countrie s[46, 48, 49]. However, it should be regarded as critical time to mobilize people who use drugs for prevention efforts—from transitioning as well as HIV and viral hepatitis prevention services.

Regarding the first type of drug that was used, while alcohol was the commonest drug first used among PWID in the study area (89.6%), the use of heroin (4.0%) and cocaine (20.8%) was worth noting. When compared to the drugs that were in current use, the same study established that brown heroin, white heroin, and cocaine were being injected by 44.8%, 30.4%, and 16.0% respectively of the PWID in the past 4 weeks [16]. Studies elsewhere have found out that heroin increased the chance of transition into premature or early regular injection [46], and that the risk of transition to injection among heroin users was higher compared to users of other types of drugs [50–52]. It has been suggested that heroin, specially injecting type, has a higher degree of dependency compared to other drugs [51]. Therefore, it is important that harm reduction services also target non-injecting drug users especially for heroin, cocaine, and alcohol as they, in addition to their role in transitioning, promote high risk sexual behavior including multiple concurrent partners and not using condoms [53, 54].

Another significant finding from this study is that sharing of needles and syringes was highly prevalent at first injection (84.8%). This was significantly higher than the PWID in the same study that had reported to be currently sharing injecting equipment (57.6%) [16]. It also corroborates findings from other international studies that the frequency of equipment sharing was higher at the first injection compared to current sharing habits, and that beginner PWID are frequently engaged in risky injecting behaviors. Studies elsewhere have established that PWID who had shared their injection equipment at the first injection were more likely to repeat this practice over the course of their injection career than those who had injected with new syringes (58.5% versus 16.5%; *p* = 0.003) [55, 56]. Therefore, in Uganda where there is limited access to injecting equipment to cover all injections [16, 57, 58], the transitioning process becomes more complex and riskier. This calls for urgent attention to prevention of transitioning and safer injection education.

Among the motivators for first injection this study, like others elsewhere, underscores the importance of peer influence cited by 47.2% of the participants. Other studies have also established that peers and social networks make transitioning acceptable and appealing [48, 59, 60]. As such, Uganda could consider interventions that target social networks for prevention of transitioning. Effective peer-education interventions and those targeting social networks as part of harm reduction have been implemented elsewhere requiring little support [57, 61–63].

Relatedly, the study also established that a desire to feel a greater “high” and pleasure influenced transitioning. This finding is in line with studies elsewhere that have shown that when other modes are no longer creating the desired state of happiness, people will tend to favor injecting due to the “rush” it creates [46, 64, 65]. This narrative suggests that increased transitions might be reflective of addiction and drug tolerance in the community. Most importantly though, it might be indicative of the need for drug treatment interventions including medication assisted therapy (MAT) since transitioning seems to occur to get a “better high” when drug users are probably developing some degree of tolerance to the drug.

Qualitative findings of this study also indicated that curiosity of what it means to feel the injection influenced transitioning—a finding that is similar to other studies [66, 67]. However, Guise and others have suggested that curiosity in itself is not enough to influence transitioning. They urge that it is a complex process that should “fit within the personal, socio-economic and political spheres” [68, 69].

## Limitations

Firstly, the study was geographically limited to two urban centers due to resource constraints despite the lack of similar data national wide.

Secondly, the study collected data that can be considered as sensitive. Participants could have potentially provided socially desirable responses, a feature that is common with RSAs among PWID [70].

Lastly, the analysis of the quantitative data did not consider determining the associations between transitioning and other variables.

Despite the above limitations, the results generated by the study still provide reliable and useful insights into the processes of transitioning of regular drug users into injecting drug use in Kampala and Mbale towns. Subsequent studies should consider increased geographical coverage, using audio computer-assisted self-interview (ACASI) to minimize social desirability bias, and establishing statistical associations for transitioning with other variables.

## Conclusion

Over 81% non-injecting drug users in Kampala and Mbale districts transitioned into injecting by the age of 24 with nearly 85% sharing injecting equipment at their first injection. This process does not only reproduce a population of PWID but also puts them at increased risk of HIV and HCV infection. As Uganda makes efforts to introduce and or strengthen harm reduction services, interventions targeting non-injecting drug users before they transition into injecting should be considered as a key component for HIV and HCV epidemic control efforts, and their evaluation considered in future research.

## Data Availability

The datasets used and/or analyzed during this study are available from the corresponding author on reasonable request.

## Abbreviations

FGDs: Focus group discussions
HCV: Hepatitis C virus
HIV: Human immunodeficiency virus
IDI: In-depth interviews
MARPI: Most at risk populations initiative
NSP: Needle and syringe program
PWID: People who inject drugs
TASO: The AIDS Support Organization
